# Instantaneous Observability of Tightly Coupled SINS/GPS during Maneuvers

**DOI:** 10.3390/s16060765

**Published:** 2016-05-27

**Authors:** Junxiang Jiang, Fei Yu, Haiyu Lan, Qianhui Dong

**Affiliations:** 1 College of Automation, Harbin Engineering University, Harbin 150001, China; 15045652295@163.com (J.J.); yufei@hrbeu.edu.cn (F.Y.); 2 Department of Gematics Engineering, University of Calgary, Calgary, AB T2N 1N4, Canada; hlan@ucalgary.ca; 3 College of Science, Harbin Engineering University, Harbin 150001, China

**Keywords:** SINS, GPS, instantaneous observability matrix (IOM), reconstructed psi-angle model, translational maneuver, angle maneuver, three-channel system, two-channel system

## Abstract

The tightly coupled strapdown inertial navigation system (SINS)/global position system (GPS) has been widely used. The system observability determines whether the system state can be estimated by a filter efficiently or not. In this paper, the observability analysis of a two-channel and a three-channel tightly coupled SINS/GPS are performed, respectively, during arbitrary translational maneuvers and angle maneuvers, where the translational maneuver and angle maneuver are modeled. A novel instantaneous observability matrix (IOM) based on a reconstructed psi-angle model is proposed to make the theoretical analysis simpler, which starts from the observability definition directly. Based on the IOM, a series of theoretical analysis are performed. Analysis results show that almost all kinds of translational maneuver and angle maneuver can make a three-channel system instantaneously observable, but there is no one translational maneuver or angle maneuver can make a two-channel system instantaneously observable. The system’s performance is investigated when the system is not instantaneously observable. A series of simulation studies based on EKF are performed to confirm the analytic conclusions.

## 1. Introduction

The system observability is an important property of a dynamic system. The observability determines whether a system state can be estimated by filters or not. It is well known that the observability would be improved during maneuvers [1,2,3,4,5]. The reason is speculated that the system state elements that cannot be observed at a low-dynamic environment are stimulated by time-varying nature of the error model. In theory, it is very difficult to analyze the observability of a time-varying system.

In an integrated strapdown inertial navigation system (SINS)/global position system (GPS), the property of the SINS and GPS is complementary. The SINS is a self-contained system and can provide continuous available navigation information at a relative high update rate. The measurements provided by the GPS receiver are used for the information fusion process to prevent the growth of navigation errors with time. Moreover, the biases of the inertial measurement unit (IMU) can be estimated, which will improve the inertial navigation performance during the periods when the satellite signals are jammed [6,7].

In a loosely coupled system, the position and velocity information provided by a GPS receiver are used. In a tightly coupled system, the data fusion algorithm processes the pseudorange and deltarange measurements provided by a GPS receiver directly. The system state consists of SINS errors, *i.e.*, position errors, velocity errors, attitude errors, and biases of the IMU. After finishing the initial alignment of a SINS, the system errors are small. A linear psi-angle model is well suited for describing the SINS error propagation [7], while the pseudorange and deltarange measurement models are still nonlinear.

There are many kinds of nonlinear filters that can be applied for the data fusion progress of pseudorange and deltarange measurements with SINS information. However, the measurement models are either very complex (e.g., Extended Kalman Filter (EKF)) or do not existent (e.g., Unscented Kalman Filter (UKF); Cubature Kalman Filter (CKF)). Compared with a loosely coupled system whose measurement model is a linear time-invariant system, this property makes it difficult to analyze the effect of maneuvers on the observability of a tightly coupled system.

In [1,2], the observability of an integrated navigation system is analyzed from a global perspective. However, such a method is very complex. Moreover, maneuvers are performed in a short time interval, we are more concerned with the observability of a system during the time intervals of maneuvers. Thus, the instantaneous observability analysis of a system is performed. If a system is instantaneously observable at every time points in a time interval, the system state can be well estimated in this interval.

In [8], Goshen-Meskin, and Bar-Itzhack, presented a method for analyzing the observability of a nonlinear system which can be modeled as a piece-wise constant system (PWCS). A stripped observability matrix (SOM) was introduced for simplifying the observability analysis. Based on this, in reference [9], the motion consisted of several different predetermined maneuvers, and then the time-varying system was modeled as a PWCS. Then the observability analysis of the in-flight alignment is performed. However, the method is not suited for a tightly coupled system, because the measurement matrix of a tightly coupled SINS/GPS is either complex or not existent at all [10,11]. Likewise, references [3,4,5,12,13,14] also need a measurement matrix.

In [15], the covariance matrix of a filter is used to analyze the observability. However, this covariance matrix is not equal to the actual covariance matrix, and the covariance matrix of a EKF is even not “consistent” with the actual covariance matrix [10,11]. Thus, this method is not suitable for a tightly coupled SINS/GPS. In addition, the observability should be the inherent property of a dynamic system, and the observability analysis should be performed on the system model rather than others.

References [3,4,5,16] investigate general observability properties during a class of vehicle motions, but those maneuvers are relatively simple, and even do not conform to practical motions. In this paper, an arbitrary translational maneuver and an arbitrary angle maneuver are modeled by the method used in SINS’s mechanization, which are closer to practical motions. Based on the IOM, we analyze the instantaneous observability of a three/two-channel system during an arbitrary translational/angle maneuver, and investigate the filter’s performance when a system is not instantaneously observable. Our contribution can be briefly divided into two parts:

(1) A novel instantaneous observability matrix (IOM) based on a reconstructed psi-angle model is proposed.
(2) An arbitrary translational/angle maneuver is modeled in a sufficient small time interval; this idea is roused by strapdown inertial navigation system mechanization.

A series of simulation studies based on EKF are performed to confirm the theoretical analysis conclusions.

This paper is organized as follows: Section 2 reconstructs a psi-angle model; Section 3 proposes a novel instantaneous observability matrix based on the reconstructed psi-angle model; Section 4 models an arbitrary translational/angle maneuver in a sufficient small time interval and analyzes the instantaneous observability of a three/two channel system during maneuvers; Section 5 carries out extensive simulation studies for verifying the validity of the theoretical analyses; and conclusions are made in Section 6.

## 2. The Reconstructed Model

As the basis of observability analysis, the reconstructed psi-angle error model of SINS must be rigorously derived. After the initial alignment process, the magnitudes of the system error state elements are small; thus, a linear psi-angle model is well qualified for describing error propagation of a SINS. In order to prevent the error growth of an unaided SINS, the IMU sensors’ biases are modeled. The choice of an appropriate model for sensors’ bias is dependent on the IMU that is used [6]. In this paper, the biases of IMU are modeled as random constants. Thus, the error model of a SINS is given as follows.

(1a) $$\delta {\dot{r}}^{c}=\;\delta v^{c}-[\omega_{\text{ec}}^{c}\times ]\delta r^{c}$$

(1b) $$\begin{array}{ll} \delta {\dot{v}}^{c}=- & [(2\omega_{\text{ie}}^{c}+\omega_{\text{ec}}^{c})\times ]\delta v^{c}+\\ & (\Gamma_{s}^{c}-[\omega_{\text{ie}}^{c}\times ][\omega_{\text{ie}}^{c}\times ])\delta r^{c}+[f^{c}\times ]\psi^{c}+T_{b}^{c}\nabla^{b} \end{array}$$

(1c) $${\dot{\psi }}^{c}\;=-[(\omega_{\text{ie}}^{c}+\omega_{\text{ec}}^{c})\times ]\psi^{c}+T_{b}^{c}\varepsilon^{b}$$

(1d) $$\begin{array}{l} {\dot{\nabla }}^{b}\;=\;0\\ \varepsilon^{b}\;=\;0 \end{array}$$

where $\Gamma_{s}^{c}$ is earth tensorial matrix of gravitation calculated by navigation computer [17].

Most GPS receivers are equipped with low-cost crystal oscillators [6], so the clock error is relatively big. We denote the receiver clock error and clock error drift as δt and δf, respectively, then, the pseudorange measurement ${\tilde{\rho }}_{i}$ from the SINS to the *i*-th satellite is modeled as Equation (2)

(2a) $$\rho_{i}(x)=\left\|r_{i,\text{sat}}^{c}-{\hat{r}}_{\text{sins}}+\delta r^{c}\right\|+c\delta t$$

(2b) $${\tilde{\rho }}_{i}={\hat{\rho }}_{i,k+1/k}+H_{\rho i}(x_{k+1}-{\hat{x}}_{k+1/k})+w_{\rho i}$$

where $r_{i,\text{sat}}^{}$ is the *i*-th satellite’s position vector relative to the Earth center, it can be received from GPS satellite ephemeris; $x={\left[\delta r^{c}\;\delta v^{c}\;\delta t\;\delta \;f\right]}^{T}$ consists of eight variables; ${\hat{\rho }}_{i,k+1/k}=\rho_{i}({\hat{x}}_{k+1/k})$, ${\hat{\rho }}_{i,k+1/k}$ is the prediction of $\rho_{i}$ at the time point k, and ${\hat{x}}_{k+1/k}$ is a part of the prediction of the system state provided by the nonlinear filter; $H_{\rho i}=({\partial \rho_{i}/\partial x}^{T}){|}_{{\hat{x}}_{k+1/k}}$ is a Jacobian associated with $\rho_{i}$; ${\hat{r}}_{\text{sins}}$ is a position vector updated by the SINS’s navigation computer; and $w_{\rho i}$ is the pseudorange measurement noise.

The deltarange measurement ${\tilde{\eta }}_{i}$ from the SINS to the *i*-th satellite, which is measured by the Doppler shift of the carrier wave, is modeled as Equation (3)

(3a) $$\eta_{i}=\frac{{\left(r_{i,\text{sat}}^{c}-{\hat{r}}_{\text{sins}}+\delta r^{c}\right)}^{T}}{\left\|r_{i,\text{sat}}^{c}-{\hat{r}}_{\text{sins}}+\delta r^{c}\right\|}(v_{i,\text{sat}}^{c}-{\hat{v}}_{\text{sins}}+\delta v^{c})+cT_{0}\delta f$$

(3b) $${\tilde{\eta }}_{i}={\hat{\eta }}_{i,k+1/k}+H_{\eta i}(x_{k+1}-{\hat{x}}_{k+1/k})+w_{\eta i}$$

where $v_{i,\text{sat}}^{c}$ is the *i*-th satellite’s velocity vector relative to the Earth, which can be received from GPS satellite ephemeris; $T_{0}$ is the period of the satellites’ electromagnetic wave signal; ${\hat{v}}_{\text{sins}}$ is the velocity vector updated by the SINS’s navigation computer; ${\hat{\eta }}_{i,k+1/k}=\eta_{i}({\hat{x}}_{k+1/k})$ is the prediction of $\eta_{i}$ at the time point k; $H_{\eta i}=({\partial \eta_{i}/\partial x}^{T}){|}_{{\hat{x}}_{k+1/k}}$ is a Jacobian associated with $\eta_{i}$; and $w_{\eta i}$ is the deltarange measurement noise.

If four or more satellites are visible, there are eight or more constraint equations composed of Equations (2b) and (3b). The measurements of a tightly coupled SINS/GPS can be rewritten as follows

(4a) $$\left[\begin{array}{c} \vdots \\ {\tilde{\rho }}_{i}-{\hat{\rho }}_{i,k+1/k}+H_{\rho i}{\hat{x}}_{k+1/k}\\ \vdots \\ {\tilde{\eta }}_{i}-{\hat{\eta }}_{i,k+1/k}+H_{\eta i}{\hat{x}}_{k+1/k} \end{array}\right]=\left[\begin{array}{c} \vdots \\ H_{\rho i}\\ \vdots \\ H_{\eta i} \end{array}\right]x_{k+1}+\left[\begin{array}{c} \vdots \\ w_{\rho i}\\ \vdots \\ w_{\eta i} \end{array}\right]=Dx_{k+1}+w_{k+1}=u_{k+1}$$

Rewriting Equation (4a) in a compact form as

(4b) $${\left(D^{T}D\right)}^{-1}D^{T}u_{k+1}=x_{k+1}+w_{k+1}^{*}$$

where *i* = 1, 2, 3, 4 (or more), D is full rank [18], and $w_{k+1}^{*}\text{=(}D^{T}D{)}^{-1}D^{T}w_{k+1}^{}$.

Equation (4b) possesses the same measurement matrix form as the measurement matrix of a loosely coupled system. Through Equation (4), we can draw a conclusion that a loosely coupled SINS/GPS is equivalent to a tightly coupled SINS/GPS essentially when the number of visible satellites is four or more. x can be calculated accurately after each measurement; in other word, x can be observed directly all the time. The analogous idea can be found in references [1,2,9,19]. Then, the filter can estimate the other elements of the system state based on the estimated values of $\delta r^{c}$ and $\delta v^{c}$. If the measurement data provided by a GPS receiver are not precise, $\delta r^{c}$ and $\delta v^{c}$ cannot be estimated precisely, and then the other elements will be contaminated.

In this paper, we analyze the instantaneous observability under the premise that four or more satellites are visible, and the measurement data provided by the GPS receiver is precise.

Rewriting Equation (1b) as

(5) $$\delta {\dot{v}}^{c}+[(2\omega_{\text{ie}}^{c}+\omega_{\text{ec}}^{c})\times ]\delta v^{c}-(\Gamma_{s}^{c}-(\omega_{\text{ie}}^{c}\times )(\omega_{\text{ie}}^{c}\times ))\delta r^{c}=[f^{c}\times ]\psi^{c}+T_{b}^{c}\nabla^{b}$$

The left side of Equation (5) is a known value to the filter. We denote the left side of Equation (6) as z, and yield

(6) $$z^{c}=[f^{c}\times ]\psi^{c}+T_{b}^{c}\nabla^{b}$$

where $z^{c}={\left[z_{1}^{c}\;z_{2}^{c}\;z_{3}^{c}\right]}^{T}$, which can be treated as a measurement.

The angular rate $\omega_{\text{ec}}^{c}$ stimulated by a motion is far less than $\omega_{\text{ie}}^{c}$; specifically, this phenomenon is more applicable for a low-speed vehicle [16], e.g., a ship or car. Thus, $\omega_{\text{ec}}^{c}\times \psi^{c}$ can be omitted in Equation (1c), the resulting simplified model is given as follows:

(7) $${\dot{\psi }}^{c}=-[\omega_{\text{ie}}^{c}\times ]\psi^{c}+T_{b}^{c}\varepsilon^{b}\;{\dot{\varepsilon }}^{b}=0_{3\times 1},\;{\dot{\nabla }}^{b}=0_{3\times 1}\;z^{c}=[f^{c}\times ]\psi^{c}+T_{b}^{c}\nabla^{b}$$

We denote the simplified error state as $y^{c}={\left[{\left(\psi^{c}\right)}^{T}\;{\left(\varepsilon^{b}\right)}^{T}\;{\left(\nabla^{b}\right)}^{T}\right]}^{T}$, rewriting Equation (7) in a compact form as

(8) $${\hat{y}}^{c}=Ay^{c},\;z^{c}\;=Cy^{c}$$

where

(9) $$A=\left[\begin{array}{ccc} -[\omega_{\text{ie}}^{c}\times ] & T_{b}^{c} & 0_{3\times 3}\\ 0_{3\times 3} & 0_{3\times 3} & 0_{3\times 3}\\ 0_{3\times 3} & 0_{3\times 3} & 0_{3\times 3} \end{array}\right]\;C=\left[\begin{array}{ccc} \;[f^{c}\times ] & \;0_{3\times 3} & T_{b}^{c}\; \end{array}\right]$$

Compared with the original model, the reconstructed model is a linear time-varying system and has a simple structure, which is easier to use for performing observability analysis.

For a two-channel system, the altitude channel is eliminated from the SINS’s mechanization equations (but a three-channel system possesses this altitude channel). It is useless to reserve the altitude channel errors (*i.e.*, $\delta v_{U}$ and $\delta r_{U}$) in the reconstructed model. Therefore, the last row of the reconstructed measurement matrix (C) is eliminated.

## 3. Instantaneous Observability Matrix

In this section, the instantaneous observability analysis of a tightly coupled SINS/GPS is introduced. Observability determines the efficiency of a nonlinear filter designed to estimate the system state [8]. Based on the reconstructed model, we design an instantaneous observability matrix (IOM) directly through the following observability definition.

**Definition 1**  [2,17]: *A system is said to be observable if for any unknown initial state $x(t_{0})$, there exists a finite $t_{1}>t_{0}$ such that the knowledge of the input and output over $\left[t_{0},t_{1}\right]$ suffices to uniquely determine the initial state $x(t_{0})$. Otherwise, the system is said to be unobservable*.

The above definition indicates that a system state is said to be observable if the initial system state $x(t_{0})$ can be determined uniquely by the measurements during the time interval $\left[t_{0},t_{1}\right]$. Meanwhile, we should also notice that the measurement data can be used to construct difference equations. If the time interval $\left[t_{0},t_{1}\right]$ is small enough, the *i*-th-order difference equation is identical to the *i*-th-order derivative (i = 1, 2, 3, ...) at the initial time $t_{0}$. Therefore, both of them involve equivalent information that can be used to derive the system initial state $x(t_{0})$. In other words, we can use the derivatives of the measurement at initial time $t_{0}$ to perform observability analysis. The initial time can be selected as any specific time point. The observability at a time point is regarded as instantaneous observability.

The derivatives of the new measurement respective to time *t* at a specific time point are listed as follows

(10) $$\;z^{c}\;=Cy^{c}\;{\dot{z}}^{c}=\dot{C}y^{c}+C{\dot{y}}^{c}=(\dot{C}+\mathrm{CA})y^{c}=N_{1}y^{c}\;{\ddot{z}}^{c}={\dot{N}}_{1}y^{c}+N_{1}{\dot{y}}^{c}=({\dot{N}}_{1}+N_{1}A)y^{c}=N_{2}y^{c}\;\vdots \;\overset{\left(k\right)}{z^{c}}={\dot{N}}_{k-1}y^{c}+N_{k-1}{\dot{y}}^{c}=({\dot{N}}_{k-1}+N_{k-1}A)y^{c}=N_{k}y^{c}$$

where $N_{k}$ describes the relation between y and the *k*-th-order derivative of z. The recursive form of $N_{k}$ is given as follows

(11) $$N_{0}=C\;N_{k}={\dot{N}}_{k-1}+N_{k-1}A$$

where k = 1, 2, 3 ⋯.

Rewriting Equation (13) in a compact form as

(12) $$Z^{c}=\Theta^{c}y^{c}$$

where

(13) $$\Theta^{c}={\left[N_{0}^{T}\;N_{1}^{T}\cdots N_{k}^{T}\right]}^{T}\;Z^{c}={\left[(z^{c}{)}^{T}\;{\left({\dot{z}}^{c}\right)}^{T}\cdots \overset{\left(i\right)}{{\left(z^{c}\right)}^{T}}\right]}^{T}$$

We denote $\Theta^{c}$ as an Instantaneous Observability Matrix (IOM), the IOM is closely related to maneuvers. Maneuvers are performed in a short time interval, so the instantaneous observability is an important property for a system. If the rank of $\Theta^{c}$ is full, y can be determined uniquely by z and its derivatives. If a system is instantaneously observable at all time points in time interval $\left[t_{0},t_{1}\right]$, y will be estimated efficiently and tends to converge in $\left[t_{0},t_{1}\right]$.

For a two-channel SINS/GPS system, the 3*i*-th, (*i* = 1, 2, 3, …) row are eliminated from $\Theta^{c}$.

## 4. Instantaneous Observability Analysis

In a sufficiently small time interval $\left[t_{0},t_{1}\right]$, the angular rate $\omega_{\text{tb}}^{t}$ and the acceleration $a^{c}$ change linearly [19], we have

(14a) $$a=a_{0}^{}+a_{1}^{}(t-t_{0}),t\in [t_{0},t_{1}]$$

(14b) $$\omega_{\text{cb}}^{}=\omega_{0}^{}+\omega_{1}^{}(t-t_{0}),t\in [t_{0},t_{1}]$$

where $\omega_{0}^{}$, $\omega_{1}^{}$, $a_{0}^{}$ and $a_{1}^{}$ are constant vectors in this small time interval $\left[t_{0},t_{1}\right]$, and the magnitude of $\omega_{0}^{}$ and $\omega_{1}^{}$ are relatively small in practice. The derivatives of $T_{b}^{c}$ and $f^{c}$ are derived as follows

(15a) $${\dot{T}}_{b}^{c}=[\omega_{\text{cb}}^{c}\times ]T_{b}^{c}\;{\ddot{T}}_{b}^{c}=[\omega_{1}^{c}\times ]T_{b}^{c}\;\overset{\left(i\right)}{T_{b}^{c}}\approx 0_{3\times 3}\;,i=3,4,5,\ldots$$

(15b) $$f^{c}=a^{c}+2[\omega_{\text{ie}}^{c}]v^{c}-g^{c}\;{\dot{f}}^{c}=a_{1}+2[\omega_{\text{ie}}^{c}]a^{c}\;{\ddot{f}}^{c}=2[\omega_{\text{ie}}^{c}]a_{1}^{c}\;{\overset{\left(i\right)}{f^{c}=0}}_{3\times 1}\;,i=3,4,5,\ldots$$

It is more clear and intuitive that those parameters of a maneuver are projected into t-frame, so the similarity transformation theorem of the skew symmetric matrix is introduced for transforming V from c-frame to t-frame, as follows

(16) $$[V^{t}\times ]=T_{c}^{t}[V^{c}\times ]T_{t}^{c}$$

where V is an arbitrary three-dimensional vector.

Substituting Equations (15a), (15b) and (16) into Equation (13) yields

(17) $$\Theta^{c}=[\begin{array}{ccc} T_{t}^{c}[f^{t}]T_{c}^{t} & 0_{3\times 3} & T_{b}^{c}\\ T_{t}^{c}[{\dot{f}}^{t}\times ]-T_{t}^{c}[f^{t}\times ][\omega_{\text{ie}}^{t}\times ]T_{c}^{t} & T_{t}^{c}[f^{t}\times ]T_{c}^{t}T_{b}^{c} & T_{t}^{c}[\omega^{t}\times ]T_{c}^{t}T_{b}^{c}\\ T_{t}^{c}[{\ddot{f}}^{t}\times ]-T_{t}^{c}[{\dot{f}}^{t}\times ][\omega_{\text{ie}}^{t}\times ]T_{c}^{t} & 2T_{t}^{c}[{\dot{f}}^{t}\times ]T_{c}^{t}-T_{t}^{c}[f^{t}\times ][\omega_{\text{ie}}^{t}\times ]T_{c}^{t}+T_{t}^{c}[f^{t}\times ][\omega^{t}\times ]T_{c}^{t}T_{b}^{c} & T_{t}^{c}[\omega_{1}^{t}\times ]T_{c}^{t}T_{b}^{c}\\ 0_{3\times 3} & 3T_{t}^{c}[{\ddot{f}}^{t}\times ]T_{c}^{t}-3T_{t}^{c}[{\dot{f}}^{t}\times ][\omega_{\text{ie}}^{t}\times ]T_{c}^{t}+3T_{t}^{c}[{\dot{f}}^{t}\times ][\omega^{t}\times ]T_{c}^{t}T_{b}^{c} & 0_{3\times 3} \end{array}]$$

Substituting Equation (17) into Equation (12) yields

(18) $$Z^{c}=\Theta^{c}y^{c}=\left[\begin{array}{ccc} T_{t}^{c} & 0_{3\times 3} & 0_{3\times 3}\\ 0_{3\times 3} & T_{t}^{c} & 0_{3\times 3}\\ 0_{3\times 3} & 0_{3\times 3} & T_{t}^{c} \end{array}\right]\Theta^{t}\left[\begin{array}{ccc} T_{c}^{t} & 0_{3\times 3} & 0_{3\times 3}\\ 0_{3\times 3} & T_{b}^{t} & 0_{3\times 3}\\ 0_{3\times 3} & 0_{3\times 3} & T_{b}^{t} \end{array}\right]y^{c}$$

Rewriting Equation (18)

(19) $$Z^{t}=\Theta^{t}y^{t}$$

where

(20a) $$Z^{t}={\left[(z^{t}{)}^{T}\;{\left({\dot{z}}^{t}\right)}^{T}\cdots \overset{\left(i\right)}{{\left(z^{t}\right)}^{T}}\right]}^{T}\;\;z^{t}\;={\left[z_{1}\;z_{2}\;z_{3}\right]}^{T}$$

and

(20b) $$y^{t}\;={\left[{\left(\psi^{t}\right)}^{T}\;{\left(\varepsilon^{t}\right)}^{T}\;{\left(\nabla^{t}\right)}^{T}\right]}^{T}\;\nabla^{t}=T_{b}^{t}\nabla^{b}={\left[\nabla_{E}\;\nabla_{N}\;\nabla_{U}\right]}^{T}\;\varepsilon^{T}\;=T_{b}^{t}\varepsilon^{b}={\left[\varepsilon_{E}\;\varepsilon_{N}\;\varepsilon_{U}\right]}^{T}$$

and

(21) $$\Theta^{t}=\left[\begin{array}{ccc} \left[f^{t}\right] & 0_{3\times 3} & I_{3\times 3}\\ \left[{\dot{f}}^{t}\times ]-[f^{t}\times ][\omega_{\text{ie}}^{t}\times \right] & \left[f^{t}\times \right] & \left[\omega^{t}\times \right]\\ \left[{\ddot{f}}^{t}\times ]-[{\dot{f}}^{t}\times ][\omega_{\text{ie}}^{t}\times \right] & 2[{\dot{f}}^{t}\times ]-[f^{t}\times ][\omega_{\text{ie}}^{t}\times ]+[f^{t}\times ][\omega \times ] & \left[\omega_{1}^{t}\times \right]\\ 0_{3\times 3} & 3[{\ddot{f}}^{t}\times ]-3[{\dot{f}}^{t}\times ][\omega_{\text{ie}}^{t}\times ]+3[{\dot{f}}^{t}\times ][\omega \times ]+[f^{t}\times ][\omega_{1}\times ] & 0_{3\times 3} \end{array}\right]$$

Equation (17) is equivalent to Equation (21), but Equation (21) has more concise form; thus, it is suited to be used for analyzing the instantaneous observability.

### 4.1. Stationary or Constant Velocity

When a vehicle is stationary or is kept constant velocity motion, and the attitude does not change, we have

(22) $$f=-g,\overset{\left(i\right)}{f^{}}=0_{3\times 1},\omega_{0}^{}=0_{3\times 1},\omega_{1}^{}=0_{3\times 1},i=1,2,3,\cdots$$

Substituting Equation (22) into Equation (21) yields

(23) $$\overset{\text{sta}/\text{conv}\_v}{\underset{\text{three}-\mathrm{channel}}{\Theta^{t}}}=\left[\begin{array}{ccc} -[g^{t}\times ] & 0_{3\times 3} & I_{3\times 3}\\ \left[g^{t}\times ][\omega_{\text{ie}}^{t}\times \right] & -[g^{t}\times ] & 0_{3\times 3}\\ 0_{3\times 3} & \left[g^{t}\times ][\omega_{\text{ie}}^{t}\times \right] & 0_{3\times 3} \end{array}\right]$$

Performing row and column elementary operations on Equation (23) yields $\text{rank}\left(\overset{\text{sta}/\text{conv}\_\text{v}}{\underset{\text{three}-\mathrm{channel}}{\Theta^{t}}}\right)=7<9$.

In this case, the system is not instantaneously observable at all time points, the simplified error state y can not be determined uniquely by z all the time; thus, y cannot be estimated accurately by a filter.

It is useful to analyze the performance of a system in a not instantaneously observable time interval. Substituting Equation (23) into Equation (19) yields

(24a) $$\psi_{E}=\;\frac{1}{g}z_{2}-\frac{1}{g}\nabla_{N}$$

(24b) $$\psi_{N}=-\frac{1}{g}z_{1}+\frac{1}{g}\nabla_{E}$$

(24c) $$\psi_{U}=-\frac{\tan\varphi }{g}z_{1}-\frac{1}{g\Omega \cos\varphi }{\dot{z}}_{2}+\frac{2}{g\Omega^{2}\sin2\varphi }{\ddot{z}}_{1}+\frac{\tan\varphi }{g}\nabla_{E}$$

(24d) $$\varepsilon_{E}\;=\;\frac{1}{g\Omega \sin\varphi }{\ddot{z}}_{1}$$

(24e) $$\varepsilon_{N}\;=\;\frac{\Omega \sin\varphi }{g}z_{2}-\frac{1}{g}{\dot{z}}_{1}-\frac{\Omega \sin\varphi }{g}\nabla_{N}$$

(24f) $$\varepsilon_{U}\;=\;(\Omega \sin\varphi \tan\varphi )z_{2}-\tan\varphi {\dot{z}}_{1}-\frac{1}{g\Omega \cos\varphi }{\ddot{z}}_{2}-(\Omega \sin\varphi \tan\varphi )\nabla_{N}$$

(24g) $$\nabla_{U}=\;z_{3}$$

Herein, $\nabla_{U}$ is only coupled with the measurement z and its derivatives, so it can be estimated efficiently. And, $\varepsilon_{E}$ is only coupled with ${\ddot{z}}_{1}$, but the coupling coefficient gΩsinφ is too small, so $\varepsilon_{E}$ can not be estimated effificenly. $\psi_{E}$, $\psi_{N}$, $\psi_{U}$, $\varepsilon_{N}$, $\varepsilon_{U}$, $\nabla_{E}$ and $\nabla_{N}$ are coupled with each other, those terms can not be distinguished efficiently.

For a two-channel system, the rank of its IOM is 6, and $\nabla_{U}$ is not coupled with measurement and other terms, so $\nabla_{U}$ cannot be observed. Other terms have the same form with Equation (24).

### 4.2. Maneuvers

It is very difficult to analyze the instantaneous observability of a system during arbitrary maneuvers. In this paper, the observability analysises during angle maneuvers and translational maneuvers are performed, respectively. This section shows why most kinds of translational/angle maneuver can make a system instantaneously observable and finds the exceptions of translational/angle maneuvers that cannot make a system instantaneously observable. First, we present a lemma, which is used later.

**Lemma 1**:  F *is a* n×n *invertible matrix,* G *is a* m×m *matrix,* E *is a* n×m *matrix,* H *is a* m×n *matrix,* m,n=1,2,3,…*, there exist*

(25) $$n+rank(G-HF^{-1}E)=rank\left(\left[\begin{array}{ll} F & E\\ H & G \end{array}\right]\right)$$

**Proof of Lemma 1:**  Because F is a n×n invertible matrix, we have

$$\left[\begin{array}{cc} I_{n\times n} & 0_{n\times m}\\ -HF^{-1} & I_{m\times m} \end{array}\right]\left[\begin{array}{cc} F & E\\ H & G \end{array}\right]\left[\begin{array}{cc} F & 0_{n\times m}\\ 0 & I_{m\times m} \end{array}\right]=\left[\begin{array}{cc} I_{n\times n} & E\\ 0 & G-HF^{-1}E \end{array}\right]$$

and

$$\begin{array}{ll} rank\left(\left[\begin{array}{ll} F & E\\ H & G \end{array}\right]\right) & =rank\left(\left[\begin{array}{cc} I & 0\\ -HF^{-1} & I \end{array}\right]\left[\begin{array}{cc} F & E\\ H & G \end{array}\right]\left[\begin{array}{cc} F & 0\\ 0 & I \end{array}\right]\right)=rank\left(\left[\begin{array}{cc} I & E\\ 0 & G-HF^{-1}E \end{array}\right]\right)\\ & =rank(F)+rank(G-HF^{-1}E)=n+rank(G-HF^{-1}E) \end{array}$$

Therfore, Lemma 1 is true.

According to Lemma 1, we have

(26) $$rank(\Theta^{t})=3+rank(\Theta_{\text{sub}}^{t})$$

where

(27) $$\Theta_{\text{sub}}^{t}=\left[\begin{array}{cc} \left[{\dot{f}}^{t}]-[f^{t}][\omega_{\text{ie}}^{t}]-[\omega^{t}][f^{t}\right] & \left[f^{t}\times \right]\\ \left[{\ddot{f}}^{t}]-[{\dot{f}}^{t}][\omega_{\text{ie}}^{t}]-[\omega_{1}^{t}][f^{t}\right] & 2[{\dot{f}}^{t}]-[f^{t}][\omega_{\text{ie}}^{t}]+[f^{t}][\omega^{t}]\\ 0 & 3[{\ddot{f}}^{t}]-3[{\dot{f}}^{t}][\omega_{\text{ie}}^{t}]+3[{\dot{f}}^{t}][\omega^{t}]+[f^{c}][\omega_{1}^{t}] \end{array}\right]$$

#### 4.2.1. Angle Maneuver

In this case, the acceleration is very small and can be neglected, for example, a SINS spins on a spot, we have

(28) $$a\approx 0_{3\times 1},\;f\approx -g$$

Substituting Equations (14b) and (28) into Equation (21) yields

(29) $$\overset{\text{angle}-\text{maneu}}{\underset{\text{three}-\text{channel}}{\Theta_{\text{sub}}^{t}}}=\left[\begin{array}{cc} \left[g^{t}][\omega_{\text{ie}}^{t}]+[\omega^{t}][g^{t}\right] & -[g^{t}]\\ \left[\omega_{1}^{t}][g^{t}\right] & \left[g^{t}][\omega_{\text{ie}}^{t}]-[g^{t}][\omega^{t}\right]\\ 0 & -[g^{t}][\omega_{1}^{t}] \end{array}\right]$$

The rank of Equation (29) is investigated by checking its null space. If the dimension of its null space is not zero, the system is not instantaneously observable.

Let $Y={\left[{\left(Y_{1}\right)}^{T}\;{\left(Y_{2}\right)}^{T}\right]}^{T}$ be an element of the null space of Equation (29), we have

(30) $$\overset{\text{angle}-\text{maneu}}{\underset{\text{three}-\text{channel}}{\Theta_{\text{sub}}^{t}}}Y=0$$

(1) **Assuming:** $\omega_{1}^{t}\neq 0_{3\times 1}$ and $\omega_{1}^{t}$ is neither perpendicular nor parallel to $g^{t}$, this assumption is valid in practice

Substituting Equation (29) into Equation (30) yields

(31a) $$0_{3\times 1}=g^{t}\times Y_{2}$$

(31b) $$0_{3\times 1}=g^{t}\times (\omega_{1}^{t}\times Y_{2})$$

(31c) $$0_{3\times 1}=\omega_{1}^{t}\times (g^{t}\times Y_{1})$$

(31d) $$0_{3\times 1}=g^{t}\times (\omega_{\text{ie}}^{t}\times Y_{1})+\omega_{}^{t}\times (g^{t}\times Y_{1})$$

It is inferred from Equation (31a) that

(32) $$Y_{2}=b_{2}g^{t},\;b_{2}\in ℛ$$

Substituting Equation (32) into Equation (31b) yields

(33) $$0_{3\times 1}=b_{2}g^{t}\times (\omega_{1}^{t}\times g^{t})$$

Thus, $b_{2}=0$ and $Y_{2}=0_{3\times 1}$.

If $Y_{1}\neq 0_{3\times 1}$, Equation (31c) implies that $Y_{1}=b_{1}g^{t}$, $b_{1}\in ℛ$ or $\omega_{1}^{t}=b_{1}^{*}Y_{1}\times g^{t}$, $b_{1}^{*}\neq 0$, $b_{1}^{*}\in ℛ$.

(a) If $Y_{1}=b_{1}g^{t}$, substituting it into Equation (31d), we have

(34) $$0_{3\times 1}=b_{1}g^{t}\times (\omega_{\text{ie}}^{t}\times g^{t})$$

Thus, $b_{1}=0$ and $Y_{1}=0_{3\times 1}$.

(b) If $\omega_{1}^{t}=b_{1}^{*}g^{t}\times Y_{1},b_{1}^{*}\neq 0,b_{1}^{*}\in ℛ$, substituting it into Equation (31d), we have

(35) $$0_{3\times 1}=g^{t}\times (\omega_{\text{ie}}^{t}\times Y_{1})+\omega_{}^{t}\times (g^{t}\times Y_{1})$$

where $g^{t}\times (\omega_{\text{ie}}^{t}\times Y_{1})$ is parallel to plane-($\omega_{\text{ie}}^{t},Y_{1}$) and perpendicular to $g^{t}$; $\omega_{}^{t}\times (g^{t}\times Y_{1})$ is parallel to plane-($g^{t},Y_{1}$) and perpendicular to $\omega_{}^{t}$;plane-($\omega_{\text{ie}}^{t},Y_{1}$) and plane-($g^{t},Y_{1}$) intersect on $Y_{1}$. If Equation (35) is valid, $g^{t}\times (\omega_{\text{ie}}^{t}\times Y_{1})$ is parallel to $\omega_{}^{t}\times (g^{t}\times Y_{1})$, then, $g^{t}\times (\omega_{\text{ie}}^{t}\times Y_{1})$, $\omega_{}^{t}\times (g^{t}\times Y_{1})$ and $Y_{1}$ are parallel to each other. Then, we have $Y_{1}$ is perpendicular to $g^{t}$ and $\omega_{}^{t}$, thus, $Y_{1}=b_{1}^{**}g^{t}\times \omega_{}^{t}$, $b_{1}^{**}\neq 0$, $b_{1}^{**}\in ℛ$. Combining $Y_{1}$ with $\omega_{1}^{t}=b_{1}^{*}g^{t}\times Y_{1}$, we have

(36) $$\omega_{1}=b_{1}^{*}b_{1}^{**}g^{t}\times (g^{t}\times \omega_{}^{t})$$

Equation (36) implies that $\omega_{1}^{t}$ is perpendicular to $g^{t}$, this result is contradict the assumption (1). Therefore, $\omega_{1}^{t}=b_{1}^{*}g^{t}\times Y_{1},b_{1}^{*}\neq 0,b_{1}^{*}\in ℛ$ is not valid, thus $Y_{1}=0_{3\times 1}$. The dimension of the null space of Equation (29) is zero, the rank of Equation (29) is full, and the system is instantaneously observable at all time points.

(2) **Assuming:** $\omega_{1}^{t}\neq 0_{3\times 1}$ and $\omega_{1}^{t}⊥g^{t}$

Substituting Equation (29) into Equation (30) yields

(37a) $$0_{3\times 1}=g^{t}\times Y_{2}$$

(37b) $$0_{3\times 1}=g^{t}\times (\omega_{1}^{t}\times Y_{2})$$

(37c) $$0_{3\times 1}=\Theta_{11}Y_{1}=([g^{t}][\omega_{\text{ie}}^{t}]+[\omega^{t}][g^{t}])Y_{1}$$

(37d) $$0_{3\times 1}=\Theta_{21}Y_{1}=[\omega_{1}^{t}][g^{t}]Y_{1}$$

It is inferred from Equations (37a) and (37b) that $Y_{2}=0_{3\times 1}$. Because of $\omega_{1}^{t}⊥g^{t}$, $\omega^{t}$ is variable and not parallel to $g^{t}$ at almost all time points. We set

(38) $$\omega^{t}={\left[\omega_{E}^{t}\;\omega_{N}^{t}\;\omega_{U}^{t}\right]}^{T},\;\omega_{1}^{t}={\left[\omega_{1,E}^{t}\;\omega_{1,N}^{t}\;0\right]}^{T}$$

where $\omega_{E}^{T}$ and $\omega_{N}^{T}$ should not be zero at the same time. $\omega_{1,E}^{t}$ and $\omega_{1,N}^{t}$ also should not be zero at the same time. Substituting Equation (38) into Equations (37c) and (37d) yields

(39) $$\Theta_{11}=[\begin{array}{ccc} g(\Omega \sin\varphi +\omega_{U}^{t}) & 0 & 0\\ 0 & g(\Omega \sin\varphi +\omega_{U}^{t}) & -g\Omega \cos\varphi \\ -g\omega_{E}^{t} & -g\omega_{N}^{t} & 0 \end{array}],\;\Theta_{21}=\left[\begin{array}{ccc} 0 & 0 & 0\\ 0 & 0 & 0\\ -g\omega_{1,E}^{t} & g\omega_{1,N}^{t} & 0 \end{array}\right]$$

We have

(40) $$\left[\begin{array}{l} \Theta_{11}\\ \Theta_{21} \end{array}\right]Y_{1}=\Theta^{*}Y_{1}=0_{6\times 1}$$

The rank of $\Theta^{*}$ is equivalent to the rank of $\Theta^{**}$ in Equation (41), as follows

(41) $$\Theta^{**}=\left[\begin{array}{cc} {Ι}_{2} & 0\\ 0 & \omega_{N}^{t}\\ 0 & \omega_{1,N}^{t} \end{array}\right]$$

If $\omega_{N}^{t}$ and $\omega_{1,N}^{t}$ are equivalent to zero at the same time, *i.e.*, $\omega_{N}^{t}=\omega_{1,N}^{t}=0$, we have

(42) $$rank(\Theta^{*})<3$$

the system is not instantaneously observable at all time points, in this case, $\omega^{t}$ and $\omega_{1}^{t}$ have the following form

(43) $$\omega^{t}={\left[\omega_{E}^{t}\;0\;\omega_{U}^{t}\right]}^{T},\;\omega_{1}^{t}={\left[\omega_{1,E}^{t}\;0\;0\right]}^{T}$$

In the other cases, the system is instantaneously observable.

(3) **Assuming:** $\omega_{1}^{t}\neq 0_{3\times 1}$ and $\omega_{1}^{t}$ is parallel to $g^{t}$, we set $\omega_{1}^{t}=b_{1}g^{t}$, $b_{1}\neq 0$, $b_{1}\in ℛ$

Substituting Equation (29) into Equation (30) yields

(44a) $$0_{3\times 1}=g^{t}\times ((\omega_{\text{ie}}^{t}-\omega^{t})\times Y_{2})$$

(44b) $$0_{3\times 1}=b_{1}g^{t}\times (g^{t}\times Y_{1})$$

(44c) $$0_{3\times 1}=g^{t}\times (\omega_{\text{ie}}^{t}\times Y_{1})+\omega_{}^{t}\times (g^{t}\times Y_{1})$$

$\omega^{t}$ is variable and not equivalent to $\omega_{\text{ie}}^{t}$ at almost any time points. It is inferred from Equation (44a) that $Y_{2}=0_{3\times 1}$.

If $Y_{1}\neq 0_{3\times 1}$, then, Equation (44b) implies that $Y_{1}=b_{1}^{*}g^{t}$, $b_{1}^{*}\neq 0$, $b_{1}^{*}\in ℛ$, substituting it into Equation (44c), we have

(45) $$0_{3\times 1}=b_{1}^{*}g^{t}\times (\omega_{\text{ie}}^{t}\times g^{t})$$

Equation (45) is invalid; therefore, $Y_{1}=0_{3\times 1}$, and the system is instantaneously observable at all time points.

(4) **Assuming:** $\omega_{1}^{t}=0_{3\times 1}$, we have

(46) $$\overset{\text{angle}-\text{maneu}}{\underset{\text{three}-\text{channel}}{\Theta_{\text{sub}}^{t}}}=\left[\begin{array}{cc} \left[g^{t}][\omega_{\text{ie}}^{t}]+[\omega_{0}^{t}][g^{t}\right] & -[g^{t}]\\ 0_{3\times 3} & \left[g^{t}][\omega_{\text{ie}}^{t}]-[g^{t}][\omega_{0}^{t}\right]\\ 0_{3\times 3} & 0_{3\times 3} \end{array}\right]$$

Substituting Equation (46) into Equation (30) yields

(47) $$0_{3\times 1}=g^{t}\times Y_{2}$$

(48) $$0_{3\times 1}=g^{t}\times ((\omega_{\text{ie}}^{t}-\omega_{0}^{t})\times Y_{2})$$

(49) $$0_{3\times 1}=\Theta_{11}Y_{1}=([g^{t}][\omega_{\text{ie}}^{t}]+[\omega_{0}^{t}][g^{t}])Y_{1}$$

If $\omega_{0}^{t}=\omega_{\text{ie}}^{t}$, there exists $Y_{2}=b_{1}g^{t}$, $b_{1}\neq 0$, $b_{1}\in ℛ$. In this case, the system is not instantaneously observable all the time.

If $\omega_{0}^{t}\neq \omega_{\text{ie}}^{t}$, we set $\omega_{0}^{t}$ as follows

(50) $$\omega_{0}^{t}={\left[\omega_{0,E}^{t}\;\omega_{0,N}^{t}\;\omega_{0,U}^{t}\right]}^{T}$$

Substituting Equation (50) into Equation (49) yields

(51) $$\Theta_{11}=\left[\begin{array}{ccc} g(\Omega \sin\varphi +\omega_{0,U}^{t}) & 0 & 0\\ 0 & g(\Omega \sin\varphi +\omega_{0,U}^{t}) & -g\Omega \cos\varphi \\ -g\omega_{0,E}^{t} & -g\omega_{0,N}^{t} & 0 \end{array}\right]$$

If $\omega_{0,N}^{t}=0$, there exist $\omega^{t}={\left[\omega_{0,E}^{t}\;0\;\omega_{0,U}^{t}\right]}^{T}$, and $\text{rank}(\Theta_{11})<3$. In this case, the system is not instantaneously observable all the time. In the other cases, the system is instantaneously observable.

It is highly likely that Assumptions (2)–(4) are invalid in practice, and Assumption (1) matches the practice better. Thus, we can say that the system is instantaneously observable during angle maneuver.

We analyze the observability properties of a two-channel system under the first assumption. In this case, the rank of the IOM is 8, and this system is not instantaneously observable at all time points. $\omega^{t}$ and $\omega_{1}^{t}$ are set as follows

(52) $$\omega^{t}={\left[\omega_{E}^{t}\;\omega_{N}^{t}\;\omega_{U}^{t}\right]}^{T},\;\omega_{1}^{T}={\left[\omega_{1,E}^{t}\;\omega_{1,N}^{t}\;\omega_{1,U}^{t}\right]}^{T}$$

and

(53) $$f^{t}\approx -g^{t},\;\overset{\left(i\right)}{f^{t}}=0_{3\times 1}$$

Substituting Equations (52) and (53) into Equation (21) yields

(54a) $$\psi_{E}=\frac{1}{g}z_{2}-\frac{1}{g}\nabla_{N}$$

(54b) $$\psi_{N}=-\frac{1}{g}z_{1}+\frac{1}{g}\nabla_{E}$$

(54c) $$\psi_{U}=-\frac{\tan\varphi }{g}z_{1}-\frac{1}{g\Omega \cos\varphi }{\dot{z}}_{2}+\frac{1}{\Omega \cos\varphi }\varepsilon_{E}-\frac{\omega_{E}^{t}}{g\Omega \cos\varphi }\nabla_{U}+\frac{\Omega \sin\varphi +\omega_{U}^{t}}{g\Omega \cos\varphi }\nabla_{E}$$

(54d) $$\varepsilon_{N}=\frac{\Omega \sin\varphi }{g}z_{2}-\frac{1}{g}{\dot{z}}_{1}-\frac{\Omega \sin\varphi +\omega_{U}^{t}}{g}\nabla_{N}+\frac{\omega_{N}}{g}\nabla_{U}$$

(54e) $$\omega_{1,E}^{t}\varepsilon_{U}-\omega_{1,U}^{t}\varepsilon_{E}=\frac{1}{g}{\dddot{z}}_{1}$$

(54f) $$\omega_{1,N}^{t}\varepsilon_{U}-\omega_{1,U}^{t}\varepsilon_{N}=\frac{1}{g}{\dddot{z}}_{2}$$

(54g) $$(\Omega \sin\varphi -\omega_{U}^{t})\varepsilon_{E}+\omega_{E}^{t}\varepsilon_{U}-\frac{\omega_{1,U}^{t}}{g}\nabla_{N}+\frac{\omega_{1,N}^{t}}{g}\nabla_{U}=\frac{1}{g}{\ddot{z}}_{1}$$

(54h) $$(\Omega \sin\varphi -\omega_{U}^{t})\varepsilon_{N}-(\Omega \cos\varphi +\omega_{N}^{t})\varepsilon_{U}+\frac{\omega_{1,U}^{t}}{g}\nabla_{E}-\frac{\omega_{1,E}^{t}}{g}\nabla_{U}={\ddot{z}}_{2}$$

All elements of the system state are coupled with each other, the filter can not distiguish them, so, the system state can not be estimated accurately. All the estimated elements interact.

#### 4.2.2. Translational Maneuver

In this case, the attitude is almost unchangeable, for example, a car accelerates, decelerates, or horizontally drifts. We have

(55) $$\omega_{\text{cb}}^{}\approx 0_{3\times 1}$$

Substituting Equations (14a) and (15b) into Equation (27) yields

(56) $$\overset{\text{trans-maneu}}{\underset{\text{three-channel}}{\Theta_{\text{sub}}^{t}}}=\left[\begin{array}{cc} \left[a_{1}+2\omega_{\text{ie}}^{t}\times a]-[f^{t}][\omega_{\text{ie}}^{t}\right] & \left[f^{t}\times \right]\\ 2[\omega_{\text{ie}}^{t}\times a_{1}]-[a_{1}][\omega_{\text{ie}}^{t}] & 2[a_{1}+2\omega_{\text{ie}}^{t}\times a]-[f^{t}][\omega_{\text{ie}}^{t}]\\ 0 & 6[\omega_{\text{ie}}^{t}\times a_{1}]-3[a_{1}][\omega_{\text{ie}}^{t}] \end{array}\right]$$

Let X be an element of the null space of Equation (56), we have

(57) $$\text{X=}\left[\begin{array}{c} X_{1}\\ X_{2} \end{array}\right],\;\overset{\text{trans-maneu}}{\underset{3\text{-channel}}{\Theta_{\text{sub}}^{t}}}X=0$$

(1) **Assuming:** $a_{1}\neq 0$, $a_{1}$ is neither parallel nor perpendicular to $\omega_{\text{ie}}^{t}$. This assumption is to be satisfied practically.

Substituting Equation (56) into Equation (57) yields

(58) $$0_{3\times 1}=2(\omega_{\text{ie}}^{t}\times a_{1})\times X_{1}-a_{1}\times (\omega_{\text{ie}}^{t}\times X_{1})$$

(59) $$0_{3\times 1}=2(\omega_{\text{ie}}^{t}\times a_{1})\times X_{2}-a_{1}\times (\omega_{\text{ie}}^{t}\times X_{2})$$

Equations (58) and (59) have the same form, we just need to analysis one of them. Equation (58) can be rewritten as

(60) $$2X_{1}\times (\omega_{\text{ie}}^{t}\times a_{1})+a_{1}\times (\omega_{\text{ie}}^{t}\times X_{1})=0_{3\times 1}$$

where $X_{1}\times (\omega_{\text{ie}}^{t}\times a_{1})$ is parallel to plane-$\left(\omega_{\text{ie}}^{t},a_{1}\right)$ and perpendicular to $X_{1}$; $a_{1}\times (\omega_{\text{ie}}^{t}\times X_{1})$ is parallel to plane-$\left(\omega_{\text{ie}}^{t},X_{1}\right)$ and perpendicular to $a_{1}$; plane-$\left(\omega_{\text{ie}}^{t},a_{1}\right)$ and plane-$\left(\omega_{\text{ie}}^{t},X_{1}\right)$ intersect on $\omega_{\text{ie}}^{t}$. If Equation (60) is valid, we have $X_{1}\times (\omega_{\text{ie}}^{t}\times a_{1})$ and $a_{1}\times (\omega_{\text{ie}}^{t}\times X_{1})$ are parallel to each other, and them would be parallel to $\omega_{\text{ie}}^{t}$, then, we conclude that $\omega_{\text{ie}}^{t}$ is perpendicular to $a_{1}$. However, the verdict appears to contradict the assumption that $a_{1}$ is not perpendicular to $\omega_{\text{ie}}^{t}$. Therefore, $X_{1}\times (\omega_{\text{ie}}^{t}\times a_{1})$ and $a_{1}\times (\omega_{\text{ie}}^{t}\times X_{1})$ are not parallel to each other. Combining this with $\omega_{\text{ie}}^{t}\times a_{1}\neq 0_{3\times 1}$, $[a_{1}][\omega_{\text{ie}}^{t}]\neq 0_{3\times 3}$, we have

(61) $$X_{1}=0_{3\times 1},\;X_{2}=0_{3\times 1}$$

Under this assumption, the dimension of the null space of Equation (56) is zero, the rank of Equation (56) is full, the system is instantaneously observable at all time points.

(2) Assuming**:** $a_{1}=0$, and $a_{0}^{t}$ is neither parallel nor perpendicular to $\omega_{\text{ie}}^{t}$, we have

(62) $$\overset{\text{trans-maneu}}{\underset{\text{three-channel}}{\Theta_{\text{sub}}^{t}}}=\left[\begin{array}{cc} 2[\omega_{\text{ie}}^{t}\times a]-[f^{t}][\omega_{\text{ie}}^{t}] & \left[f^{t}\times \right]\\ 0 & 4[\omega_{\text{ie}}^{t}\times a]-[f^{t}][\omega_{\text{ie}}^{t}]\\ 0 & 0 \end{array}\right]$$

Substituting Equation (57) into Equation (62) yields

(63) $$\text{2(}\omega_{\text{ie}}^{t}\times a_{0})\times X_{2}-f^{t}\times (\omega_{\text{ie}}^{t}\times X_{2})=0_{3\times 1}$$

(64) $$\text{4(}\omega_{\text{ie}}^{t}\times a_{0})\times X_{2}-f^{t}\times (\omega_{\text{ie}}^{t}\times X_{2})=0_{3\times 1}$$

Using the analytical method, which is similar to the methods used in Equation (58) and (59), we have

(65) $$X_{1}=0_{3\times 1},\;X_{2}=0_{3\times 1}$$

Under this assumption, the dimension of the null space of Equation (60) is zero, the rank of Equation (60) is full, the system is instantaneously observable, and the system state can be distinguished and estimated efficiently by a filter.

The observability analysis of a two-channel system is performed under the above assumptions. In this case, the rank of its IOM is 8, and, the last three columns of Equation (21) are list as follows

(66) $$\overset{\text{trans-maneu}}{\underset{\text{three-channel}}{\Theta_{\text{sub}}^{t}(c_{7},c_{8},c_{9})}}=\left[\begin{array}{ccc} 1 & 0 & 0\\ \begin{array}{l} 0\\ 0\\ \vdots \\ 0 \end{array} & \begin{array}{l} 1\\ 0\\ \vdots \\ 0 \end{array} & \begin{array}{l} 0\\ 0\\ \vdots \\ 0 \end{array} \end{array}\right]$$

$\nabla_{U}$ is neither coupled with measurements nor other elements of the simplified error state. Thus, $\nabla_{U}$ can not be observed by a filter, the other elements can be distinguished and estimated accurately.

## 5. Simulations and Results

To evaluate the observability analysis conclusions in Section 4, a series of numerical simulations based on a EKF are performed in this section. The accelerometer constant bias and gyroscope constant drift is set as 10^−3^ g and 0.1°/h, respectively; all measurement noise are treated as Gaussian noise, the standard derivations of acceleration measurement noise is set as 5 × 10^−4^ g; the standard derivations of gyroscope measurement noise is set as 0.05°/h; and the standard derivations of pseudorange measurement noise and delta pseudorange measurement noise are set as 0.5 m and 0.01 m/s, respectively. The initial attitude angle error vector is [20.6265″ 22.6891″ 196.6309″].

ψ defines the attitude of p-frame relative to c-frame, δθ defines the attitude of c-frame relative to t-frame, and

(67) $$\delta \theta^{c}={\left[\delta r_{E}/r\;-\delta r_{N}/r\;-\delta r_{E}\tan\hat{\varphi }/r\right]}^{T}$$

where $r=R_{e}+h$, so the small rotation vector defines the attitude of c-frame relative to t-frame is ϕ=δθ+ψ.

### 5.1. Simulation 1: Stationary

In simulation 1, we test and investigate the performance of a filter designed for a tightly coupled SINS/GPS with a stationary condition. Simulation figures are list as follows

#### (a) Three-channnel system

**Figure 1:**
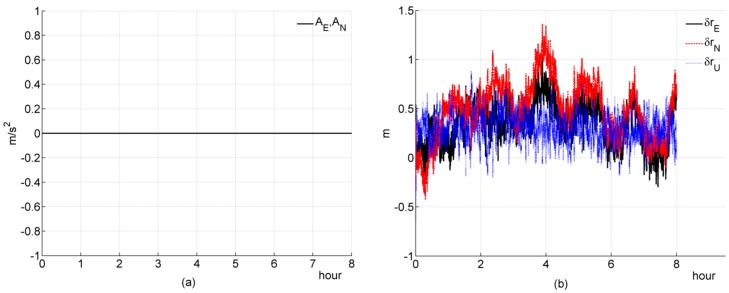

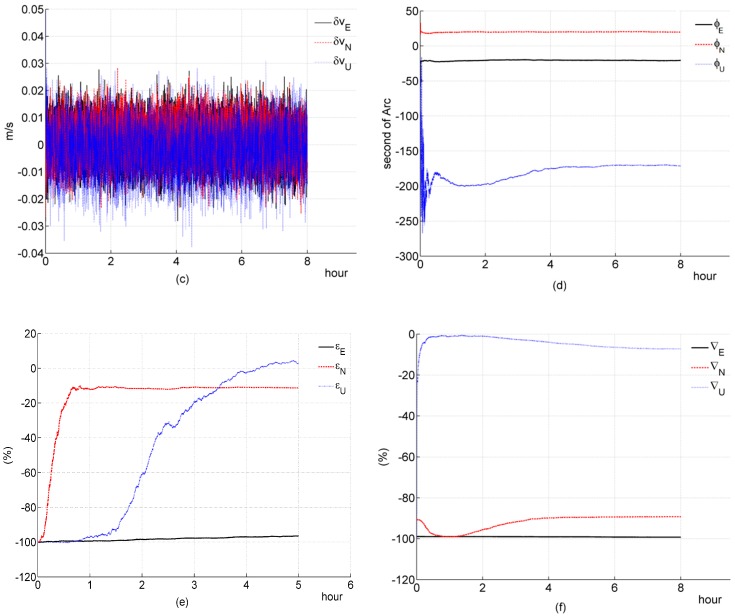
Simulation results of a three-channel system during a stationary condition. (**a**) acceleration; (**b**) deviation of estimated position error elements; (**c**) deviation of estimated velocity error elements; (**d**) deviation of estimated platform error angle; (**e**) percentage of deviation of estimated gyroscope bias; (**f**) percentage of deviation of estimated accelerometer bias.

In Figure 1e, the equivalent Easten Gyroscope Constant Drift $\varepsilon_{E}$ can not be well estimated; In Figure 1f, the equivalent Altitude Accelerometer Constant Bias $\nabla_{U}$ can be well estimated. The above two simulation results are consistent with the theoretical analysis. And, the convergence rate of $\nabla_{U}$ is very fast, this result is also consistent with the previous theoretical analysis (Section 4.2.2). It seems that the equivalent Altitude Gyroscope Constant Drift $\varepsilon_{U}$ can also be estimated by the filter, which seems to breach the previous theoretical analysis (Section 4.2.2). This is because the coefficient $\Omega \sin^{2}\varphi \nabla_{N}/\cos\varphi$ in Equation (24f) is negligibly small, on the order of 10^−8^; thus, Equation (24f) can be rewritten as follows

(68) $$\varepsilon_{U}=\frac{\Omega \sin^{2}\varphi }{\cos\varphi }z_{2}-\tan\varphi {\dot{z}}_{1}-\frac{1}{g\Omega \cos\varphi }{\ddot{z}}_{2}$$

Herein, the $\varepsilon_{U}$ is only coupled with the measurement z and its derivatives, so it can be estimated. This result also supports the previous theoretical analysis. In Equation (29e), the coefficient $\Omega \sin\varphi \nabla_{N}/g$ is negligibly small, on the order of 10^−9^; thus, Equation (29e) can be rewritten as follows

(69) $$\varepsilon_{N}=\frac{\Omega \sin\varphi }{g}z_{2}-\frac{1}{g}{\dot{z}}_{1}$$

the coefficients −1/g and Ωsinφ/g in Equation (69) are much less than the coefficients in Equation (68), so the coupling between $\varepsilon_{N}$ and $z_{2}$, ${\dot{z}}_{1}$ are much weaker, and the convergence rate of $\varepsilon_{N}$ is much slower than $\varepsilon_{U}$.

#### (b) Two-channnel system

**Figure 2:**
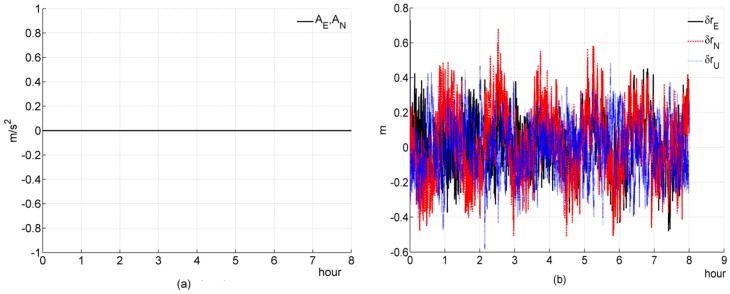

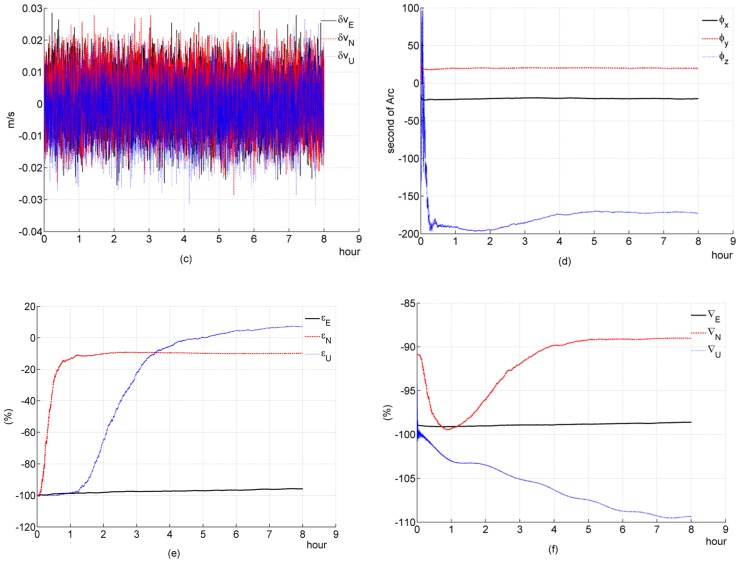
Simulation results of a two-channel system during a stationary condition. (**a**) acceleration; (**b**) deviation of estimated position error elements; (**c**) deviation of estimated velocity error elements; (**d**) deviation of estimated platform error angle; (**e**) percentage of deviation of estimated gyroscope bias; (**f**) percentage of deviation of estimated accelerometer bias.

In Figure 2f, the equivalent Altitude Accelerometer Constant Bias ($\nabla_{U}$) cannot be estimated efficiently, the other elements show on the same behaviors as the counterparts in a three-channel system. These results are consistent with the previous theoretical analysis (Section 4.2.2).

### 5.2. Simulation 2: Translational Maneuver

In simulation 2, we test and investigate its performance during a slop acceleraton maneuver. The relative parameters of this translational maneuver are listed in Table 1.

Simulation figures are listed as follows:

#### (a) Three-channnel system

**Figure 3:**
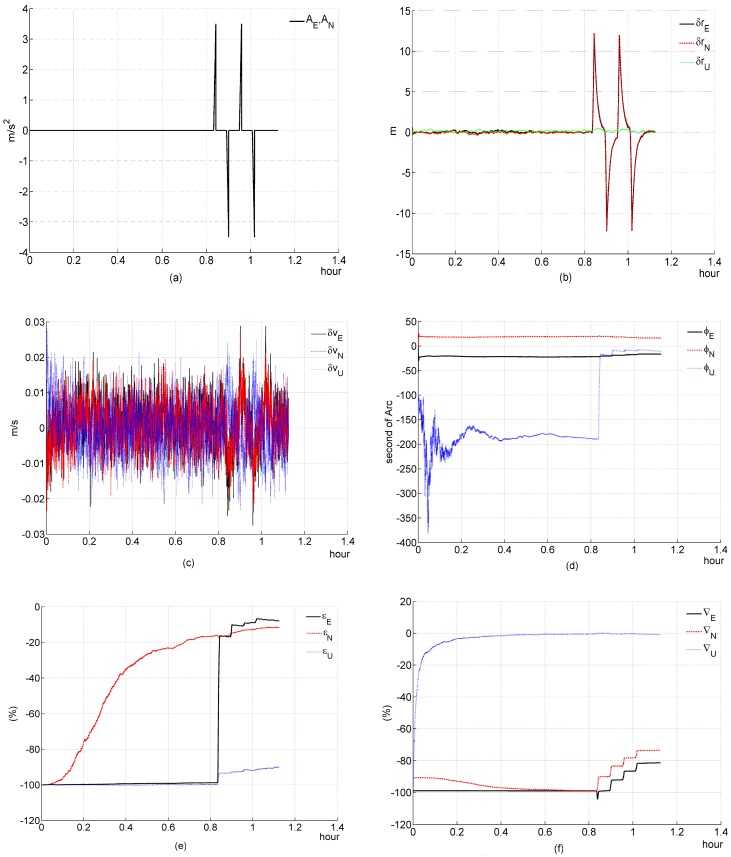
Simulation results of the three-channel system during a slop acceleration maneuver. (**a**) acceleration; (**b**) deviation of estimated position error elements; (**c**) deviation of estimated velocity error elements; (**d**) deviation of estimated platform error angle; (**e**) percentage of deviation of estimated gyroscope bias; (**f**) percentage of deviation of estimated accelerometer bias.

According to the theoretical analysis in Section 4.2.2, the system is instantaneously observable at any time point during $T_{\text{tra\_maneu}}$. All elements of the system state have been well estimated, as shown in Figure 3, and all of them only converge quickly during $T_{\text{tra\_maneu}}$. This simulation result is consistent with the theoretical analysis (Section 4.2.2).

#### (b) Two-channnel system

**Figure 4:**
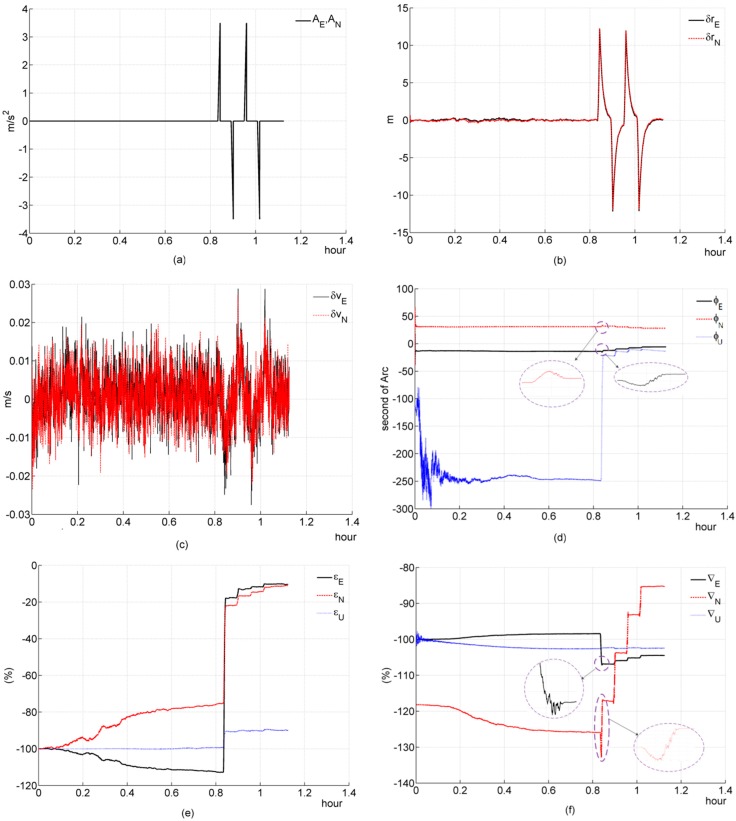
Simulation results of the two-channel system during a slop acceleration maneuver. (**a**) acceleration; (**b**) deviation of estimated position error elements; (**c**) deviation of estimated velocity error elements; (**d**) deviation of estimated platform error angle; (**e**) percentage of deviation of estimated gyroscope bias; (**f**) percentage of deviation of estimated accelerometer bias.

Figure 4f shows $\nabla_{U}$ can not be estimated efficiently, which is consistent with the theoretical analysis (Section 4.2.2). It seems that $\nabla_{E}$, $\nabla_{N}$ and $\phi_{N}$ have not been estimated well during the first translational maneuver. They just “jump” to a wrong direction. This is because the filter takes time to adjust, since the previous estimated system state is not precise, and a two-channel system has less constraint conditions than a three-channel system, which can be seen from, compared with a three-channel system, the 3*i-*th rows (*i* = 1, 2, 3, …) of a two-channel system’s IOM are eliminated. Therefore, the filter takes more time to adjust, which causes that “jump”. These simulation results are consistent with the theoretical analysis (Section 4.2.2).

### 5.3. Simulation 3: Angle Maneuver

In simulation 3, we test and investigate the system’s performance during a triangle angle velocity maneuver. It is set that the b-frame is alligned to the t-frame at the initial time point, *i.e.*, $T_{b}^{t}(t_{0})=I_{3\times 3},t_{0}=0$. The relative parameters of this angle manerver are listed in Table 2.

Simulation figures are list as follows:

#### (a) Three-channnel system

**Figure 5:**
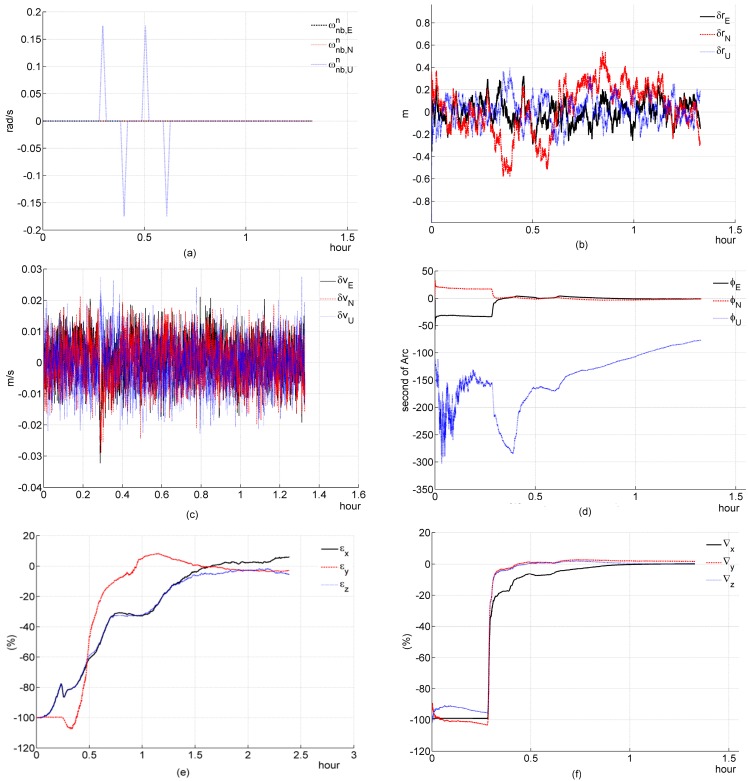
Simulation results of three-channel system during a triangle angle velocity maneuver. (**a**) angular velocity of b-frame relative to n-frame; (**b**) deviation of estimated position error elements; (**c**) deviation of estimated velocity error elements; (**d**) deviation of estimated platform error angle; (**e**) percentage of deviation of estimated gyroscope bias; (**f**) percentage of deviation of estimated accelerometer bias.

According to the theoretical analysis in Section 4.2.1, the system is instantaneously observable at any time point during $T_{\text{ang\_maneu}}$. All elements of the system state have been well estimated, as shown in Figure 5. All of them converge to truth-value during $T_{\text{ang\_maneu}}$. The simulation result is consistent with the theoretical analysis (Section 4.2.1).

#### (b) Two-channnel system

**Figure 6:**
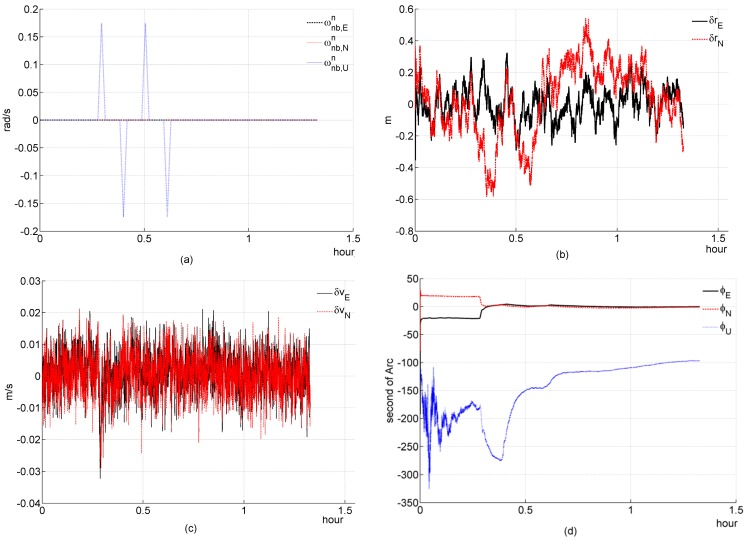

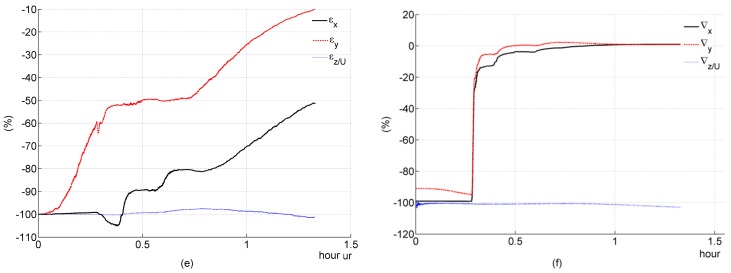
Simulation results of two-channel system during a triangle angle velocity maneuver. (**a**) angular velocity of b-frame relative to n-frame; (**b**) deviation of estimated position error elements; (**c**) deviation of estimated velocity error elements; (**d**) deviation of estimated platform error angle; (**e**) percentage of deviation of estimated gyroscope bias; (**f**) percentage of deviation of estimated accelerometer bias.

Figure 6 shows that $\varepsilon_{U}$ and $\nabla_{U}$ cannot be estimated efficiently, but the other elements can be well estimated by the filter. Theoretically, Equation (54) can be rewritten during this angle maneuver as follows

(70a) $$\psi_{E}=\frac{1}{g}z_{2}-\frac{1}{g}\nabla_{N}$$

(70b) $$\psi_{N}=-\frac{1}{g}z_{1}+\frac{1}{g}\nabla_{E}$$

(70c) $$\psi_{U}=-\frac{\tan\varphi }{g}z_{1}-\frac{1}{g\Omega \cos\varphi }{\dot{z}}_{2}-\frac{1}{\Omega \omega_{1,U}g\cos\varphi }{\dddot{z}}_{1}+\frac{\Omega \sin\varphi +\omega_{U}}{g\Omega \cos\varphi }\nabla_{E}$$

(70d) $$\varepsilon_{N}=-\frac{1}{\omega_{1,U}g}{\dddot{z}}_{2}$$

(70e) $$\varepsilon_{E}=-\frac{1}{\omega_{1,U}g}{\dddot{z}}_{1}$$

(70f) $$\nabla_{N}=-\frac{1}{\omega_{1,U}}{\ddot{z}}_{1}-\frac{\Omega \sin\varphi -\omega_{U}}{\omega_{1,U}\omega_{1,U}}{\dddot{z}}_{1}$$

(70g) $$\nabla_{E}=\frac{\Omega \sin\varphi -\omega_{U}}{\omega_{1,U}\omega_{1,U}}{\dddot{z}}_{2}+\frac{1}{\omega_{1,U}}{\ddot{z}}_{2}+\frac{g\Omega \cos\varphi }{\omega_{1,U}}\varepsilon_{U}$$

Equations (70a), (70b) and (70d)–(70f) show that $\psi_{E}$, $\psi_{N}$, $\varepsilon_{N}$, $\varepsilon_{E}$ and $\nabla_{N}$ are only relevant with the measurement and its derivatives, and that these terms can be well estimated. In Equation (70g), $g\Omega \cos\varphi \varepsilon_{U}/\omega_{1,U}$ is very small, its magnitude is on the order of 10^−7^, so it can be eliminated. Equation (70g) can be rewritten as

(71) $$\nabla_{E}=\frac{\Omega \sin\varphi -\omega_{U}}{\omega_{1,U}\omega_{1,U}}{\dddot{z}}_{2}+\frac{1}{\omega_{1,U}}{\ddot{z}}_{2}$$

Therefore, $\psi_{U}$ and $\nabla_{E}$ can be estimated efficiently. In a word, the above simulation results are consistent with the theoretical analysis (Section 4.2.1).

## 6. Conclusions

We analyze the instantaneous observability of a three/two channel tightly coupled SINS/GPS navigation system, and investigate the performance of a filter when a system is not instantaneously observable.

During a stationary mode or a constant velocity mode, the equivalent eastern/altitude gyroscope constant drift and the equivalent altitude accelerometer constant bias of a three-channel system can be estimated efficiently. However, for a two-channel system, only the equivalent eastern/altitude gyroscope constant drift can be estimated well. Almost all kinds of translational maneuver can make a three-channel system be instantaneously observable. For a two-channel system, the equivalent altitude accelerometer constant bias cannot be observed during all kinds of translational maneuver. Almost all kinds of angle maneuver can make a three-channel system be instantaneously observable. Those exceptions that cannot make a three-channel system be instantaneously observable are given in Section 4.2.2. For a two-channel system, all kinds of translational maneuver cannot make it instantaneously observable; thus, its instantaneous analysis should be made according to specific circumstance.

Based on this research, the IOM can be regarded as an efficient and proper approach for analyzing the instantaneous observability of a tightly coupled SINS/GPS during translational/angle maneuver.

## Figures and Tables

**Table 1:** The parameters of the translational maneuver

Execution Time ($T_{\text{tra\_maneu}}$)	Motion	$a_{1}$ (m/s^3^)
The other time	stationary	[0, 0, 0]
[1200 s, 1235 s], [1550 s, 1585 s],	slope	[0.1, 0.1, 0]^T^
[1375 s, 1410 s], [1725 s, 1760 s].	acceleration	−[0.1, 0.1, 0]^T^

**Table 2:** The parameters of the angle manerver.

Execution Time ($T_{\text{action}}$)	Motion	$\omega_{1}$ (rad/s^2^)
The other time	stationary	[0, 0, 0]
[1000 s, 1060 s]; [1420 s, 1480 s]; [1720 s, 1780 s]; [2140 s, 2200 s].	tri-angle velocity	[0, 0, 2.77 × 10^−3^]^T^
[1060 s, 1120 s]; [1780 s, 1840s]; [1360 s, 1420s]; [2080 s, 2140 s].		−[0, 0, 2.77 × 10^−3^]^T^

## Abbreviations

The following abbreviations are used in this manuscript:

Abbreviations:	
SINS	strapdown inertial navigation system;
GPS	global position system;
IOM	instantaneous observability matrix;
EKF	Extended Kalman Filter;
CKF	cubature Kalman filter;
UKF	Unscented Kalman filter;
symbol:	
$A,A_{1}$-frame	arbitrary coordinate frames;
$T_{A}^{A_{1}}$	direction cosine matrix that transforms a vector from its A-frame projection form to its $A_{1}$-frame projection form;
I	identity matrix;
V	arbitrary vector without specific coordinate frame designation;
$V^{A}$	column matrix with elements equal to the projection of V on A-frame axis, and $V^{A}=[V_{x}^{A}\;V_{y}^{A}\;V_{z}^{A}]$;
$\left[V^{A}\times \right]$	skew symmetric(or cross product)form of $V^{A}$, represented by the square matrix, $\left[\begin{array}{ccc} 0 & -V_{z}^{A} & V_{y}^{A}\\ V_{z}^{A} & 0 & -V_{x}^{A}\\ -V_{y}^{A} & V_{x}^{A} & 0 \end{array}\right]$, matrix product of $\left[V^{A}\times \right]$ with another A-frame vectors equals the cross product of $V^{A}$ with the vector in the A-frame;
‖V‖	norm of V;
$\omega_{AA_{1}}$	angular rate of $A_{1}$-frame relative to A-frame;
δr	the position-error vector of a SINS;
δv	the velocity-error vector of a SINS;
ψ	the attitude-error vector of a SINS;
∇	the constant-bias vector of an accelerometer;
ε	the constant-drift vector of a gyroscope;
h	altitude;
φ	latitude;
$\hat{h}$	computed altitude;
$\hat{\varphi }$	computed latitude;
$R_{e}$	earth radius;
Ω	Earth rotating rate;
${\tilde{\rho }}_{i}$	The pseudorange measurement from the SINS to the *i*-th satellite;
${\tilde{\eta }}_{i}$	The deltarange measurement from the SINS to the *i*-th satellite;
$r_{i,\text{sat}}^{}$	The *i*-th satellite’s position vector relative to earth center;
$v_{i,\text{sat}}^{}$	The *i*-th satellite’s velocity vector relative to earth;
${\hat{r}}_{\text{sins}}$	The position vector updated by navigation computer;
${\hat{v}}_{\text{sins}}$	The velocity vector updated by navigation computer;
The coordinate frames are defined as follows:	
navigation frame	the navigation frame has its z axis parallel to the upward vertical at the local Earth surface reference position location, x-axis is parallel to the EAST direction, y-axis is parallel to the NORTH direction;
t-frame	navigation frame at the true Earth surface reference position location, we denote $V^{t}$ as $V^{t}={\left[V_{E}\;V_{N}\;V_{U}\right]}^{T}$;
c-frame	navigation frame at the computed Earth surface reference position location;
b-frame	body frame;
i-frame	inertial frame;
e-frame	earth frame, it is the Earth fixed coordinate used for position location definition; its z-axis is parallel to the polar axis;
p-frame	platform frame.
